# Neonatal Pneumothorax Outcome in Preterm and Term Newborns

**DOI:** 10.3390/medicina58070965

**Published:** 2022-07-20

**Authors:** Miljana Z. Jovandaric, Svetlana J. Milenkovic, Jelena Dotlic, Ivana R. Babovic, Zorica Jestrovic, Branislav Milosevic, Miljan Culjic, Sandra Babic

**Affiliations:** 1 Department of Neonatology, Clinic for Gynecology and Obstetrics, University Clinical Center of Serbia, 11000 Belgrade, Serbia; ceca.milenkovic@yahoo.com; 2 Department of Gynecology and Obstretics, Clinic for Gynecology and Obstetrics, University Clinical Center of Serbia, 11000 Belgrade, Serbia; drenadot@gmail.com (J.D.); ivana.r.babovic@gmail.com (I.R.B.); zjestrovic@gmail.com (Z.J.); drbanemilosevic@gmail.com (B.M.); dr.chuka@gmail.com (M.C.); sandradrmilic@yahoo.com (S.B.); 3 Medical Faculty, University of Belgrade, 11000 Belgrade, Serbia

**Keywords:** newborn, therapy, pneumothorax

## Abstract

*Background and Objectives:* Pneumothorax implies the presence of air in the pleural space between the visceral and parietal pleura. The aim of this study was to investigate the incidence, clinical characteristics, risk factors, therapy and perinatal outcome in neonates with pneumothorax in a tertiary care center. *Materials and Methods:* A retrospective study based on a five-year data sample of neonates with pneumothorax was conducted in a Maternity Hospital with a tertiary NICU from 2015 to 2020. We included all neonates with pneumothorax born in our hospital and compared demographic characteristics, perinatal risk factors, anthropometric parameters, comorbidities, clinical course and method of chest drainage between term (≥37 GW) and preterm (<37 GW) neonates. *Results:* The study included 74 newborns with pneumothorax, of which 67.6% were male and 32.5% were female. The majority of women (59.5%) had no complications during pregnancy. Delivery was mainly performed via CS (68.9%). Delivery occurred on average in 34.62 ± 4.03 GW. Significantly more (*p* = 0.001) children with pneumothorax were born prematurely (*n* = 53; 71.6%) than at term (*n* = 21; 28.4%). Most of the neonates had to be treated with ATD (63.5%) and nCPAP (39.2%), but less often they were treated with surfactant (40.5%) and corticosteroids (35.1%). O_2_ therapy lasted an average of 8.89 ± 4.57 days. Significantly more (*p* = 0.001) neonates with pneumothorax had additional complications, pneumonia, sepsis, convulsions and intraventricular hemorrhage (68.9%). However, most children had a good outcome (83.8%) and were discharged from the clinic. Fatal outcomes occurred in six cases, while another six neonates had to be transferred to referral neonatal centers for further treatment and care. *Conclusion:* Significantly more children with pneumothorax were born prematurely than at term. With adequate therapy, even premature newborns can successfully recover from pneumothorax.

## 1. Introduction

Pneumothorax implies the presence of air in the pleural space between the visceral and parietal pleura [1].

With the first inspiratory breath, the newborn creates a transpulmonary pressure greater than 100 cm of water column and opens the lungs that were closed in utero. After a few breaths, this pressure normalizes and the lungs begin to function. If the transpulmonary pressure remains elevated for a long time, alveolar rupture occurs, accumulating air between the visceral and parietal pleura. The air created in the interpleural space leads to a pathological condition called neonatal pneumothorax (NP). Pneumothorax developed in this way is called spontaneous (primary, idiopathic) [2]. The reason for secondary pneumothorax in newborns can be respiratory distress syndrome (RDS), mechanical ventilation (MV), pneumonia, sepsis, aspiration of meconium, blood and amniotic fluid or congenital lung malformations [3,4].

Depending on the size of the lung collapse, pneumothorax can be partial or complete, unilateral or bilateral. A small amount of air can be asymptomatic and resorbed in the body, while the presence of a larger amount of air endangers the life of the newborn [5,6]. The incidence of NP in newborns is 1 to 2%, most often asymptomatic in 98% of cases. It is more common in premature babies, at as much as 5–7% in newborns weighing less than 1500 g [6]. The incidence of NP in Tuzla, 2/1000 [7], Oman, 2.5/1000 [8], Turkey, 1% to 2% [9] and Portugal, 1.5% in NICU [10], is less than in Saudi Arabia, 3.9% in NICU [11], Egypt, 9.1% [12], Iran, 5.8% [13] and Canada, 2.5–6.7% [14]. In a Danish study, the frequency of NP was 1.4/1000 [6], and in South India it was 1.3/1000 [15].

## 2. Aim

The aim of the study was to examine and compare the early neonatal outcomes of preterm and full-term neonates who had pneumothorax at birth and to assess which parameters may influence this outcome.

## 3. Material and Methods

A retrospective study based on a five-year data sample of neonates with pneumothorax was conducted at the Clinic for Gynecology and Obstetrics of the University Clinical Center of Serbia in Belgrade from January 2015 to December 2020. During this period, 30,378 newborns were born in our maternity hospital. In a 14-bed tertiary NICU with 3911 admissions over a 5-year period, we selected all neonates with radiographically confirmed neonatal pneumothorax (NP). Neonates with milder forms of respiratory distress syndrome probably had mild forms of undiagnosed air leakage. The study included exclusively all newborns with X-ray-confirmed pneumothorax.

The data were collected from the medical records of the newborns and their mothers. Informed consent for pneumothorax treatment was obtained from the mothers of all treated patients.

The patients were evaluated on the basis of demographic characteristics (gender, gestational age, birth weight, APGAR score at the 5th minute and need for resuscitation), perinatal (gestational disorders—gestational diabetes mellitus (GDM), hypertension, preeclampsia, HELLP syndrome, placental abruption, premature rupture of membranes (PPROM), mode of delivery, multiple pregnancies) and neonatal data (basic lung disorders—respiratory distress syndrome (RDS), pneumonia, meconium aspiration syndrome (MAS), transient tachypnea of the newborn (TTN) and comorbidities: intraventricular hemorrhage (IVH), retinopathy of prematurity (ROP), seizures, sepsis), as well as pneumothorax characteristics (laterality, age of onset, need for surfactant, oxygen therapy, nasal continuous positive airway pressure (nCPAP) and mechanical ventilation (MV) before NP, type of treatment (conservative or drainage), duration of drainage, MV and oxygen therapy after NP) and outcome.

We compared differences between term (≥37 GW) and preterm (<37 GW) neonates with NP and assessed risk factors for thoracic drainage.

### Statistical Analysis

Data were analyzed using descriptive (percentage, mean value, standard deviation—SD) and analytical statistics using SPSS 20 software. A probability value of *p* less than 0.05 was considered statistically significant. The significance of the difference between groups of neonates was examined by ANOVA and the Kruskal–Wallis χ2 test. Spearman’s correlation was applied to investigate the relationship between the estimated parameters and the neonatal outcome.

## 4. Results

Out of 30,378 newborns born in our maternity hospital, 74 developed pneumothorax (2.4/1000 newborns). The study included 74 newborns with pneumothorax, of which 67.6% were male and 32.5% were female. There were 10 twin pregnancies. The majority of women (59.5%) had no complications during pregnancy. Delivery was mainly performed via CS (68.9%). Delivery occurred on average in 34.62 ± 4.03 GW (min = 24; max = 40). Significantly more (*p* = 0.001) children with pneumothorax were born prematurely (*n* = 53; 71.6%) than at term (*n* = 21; 28.4%). Children were generally in good condition at birth (eutrophic in 62.2% and with an average Apgar score of 7.03 ± 2.28).

Most of the neonates had to be treated with ATD (63.5%) and nCPAP (39.2%), but less often they were treated with surfactant (40.5%) and corticosteroids (35.1%). O_2_ therapy lasted an average of 8.89 ± 4.57 days.

Significantly more (*p* = 0.001) neonates with pneumothorax had additional complications, pneumonia, sepsis, convulsions or intraventricular hemorrhage (68.9%). Sepsis and pneumonia were treated with antibiotic therapy. The length of antibiotic therapy was assessed by findings of elevated infection parameters, blood test results, C reactive protein (CRP) values, blood culture findings (all inoculated media remained sterile) and X-ray findings in pneumonia. Convulsions were treated with Phenobarbitone, with an initial dose of 20 mg/kg/bw, and a maintenance dose of 5 mg/kg/bw for 5 days. Intraventricular hemorrhage was controlled by echosonographic examination. However, most children had a good outcome (83.8%) and were discharged from the clinic. Mortality was recorded in six neonates (five neonates below 25 GW, and in one neonate at term due to Potter syndrome).

The frequency of tested parameters in premature neonates and newborns with pneumothorax at birth is shown in Table 1, while descriptive data of the examined children are shown in Table 2.

**Figure 1:**
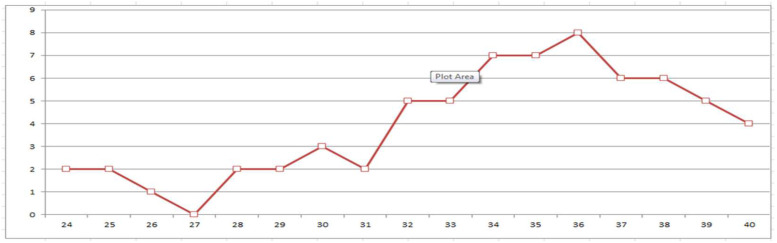
Frequency of pneumothorax by gestational weeks (GW).

It can be seen that all twin pregnancies ended before the due date. There were no significant differences between neonates with pneumothorax at birth who were born preterm and at term with regard to their sex, body development, pregnancy complications and time spent with ruptured membranes before delivery, type of delivery, side of pneumothorax, type of treatment and duration of O_2_ therapy, saturation, CRP, infections and neurological complications. In contrast, surfactant and corticosteroids were administered significantly more frequently in neonates born prematurely, as they were also more likely to have problems with oxygenation (asphyxia and RDS). Furthermore, all biometric parameters at birth as well as the Apgar scores were significantly different between preterm and term neonates. However, the final outcome of neonates with pneumothorax was similar regardless of the term of delivery. Pneumothorax most commonly occurred at 36 GW (Figure 1).

## 5. Discussion

Premature birth and respiratory distress syndrome (RDS) are the most important causes of neonatal pneumothorax. The cause of RDS is insufficient surfactant production in the lungs of a premature neonate [7]. RDS is the main reason for pneumothorax in preterm neonates. The basis of the manifestation of RDS can occur due to lung immaturity or disorders in the synthesis and function of the surfactant due to pathological processes (asphyxia, conatal pneumonia, gestational hypertension, GDM as well as preeclampsia of the pregnant woman) [16,17,18]. In our study, delivery on average occurred in the 34.62 ± 4.03 GW (min = 24; max = 40). Significantly more (*p* = 0.001) children with pneumothorax were born prematurely (*n* = 53; 71.6%) than in term (*n* = 21; 28.4%).

In a study by Vibede L. et al., the prevalence of RDS in pneumothorax was 43%, compared to 56.6% in our study. The same authors cite 52 newborns at term with pneumothorax of 48,968 live births and 71 cases of PN, giving an incidence of 0.14%, during 8 years of observation from 2006 to 2014 [6].

The higher probability of developing RDS in male neonates has been investigated in animal models, in premature male and female lambs [19]. Isak N. et al., in research on lambs, states there are higher lecithin/sphingomyelin (L/S) ratio and higher concentrations of saturated phosphatidylcholine in amniotic fluid during human pregnancy with female fetuses, indicating a greater degree of lung maturity (in terms of surfactant composition) in females. Poor respiratory adaptation can result from gender differences in the composition of the phospholipid constituents of the surfactant and its function [20,21,22,23].

Male gender dominates as a risk factor for the development of pneumothorax in most studies, which was confirmed in our study, where two-thirds of the sample with NP were male newborns (62.3% of premature babies and 81% of male babies developed pneumothorax).

In their study, Prediger B. et al. state that neonates delivered by elective cesarean section had an increased incidence of pneumothorax (2.90/1000 births), in comparison with neonates delivered by emergency cesarean (1.53/1000 births). In our study, with caesarean section, 69.8% of premature babies were born, and 66.7% of term newborns with pneumothorax. Neonates born by caesarean section (CS) often have “wet lungs” accompanied by forced breathing that can lead to pneumothorax. In neonates born with CS at term or moderately preterm (30–36 weeks), the incidence of NP and respiratory problems is significantly increased. These problems can be avoided by postponing childbirth and administering antenatal steroid therapy where appropriate and possible. Antenatal corticosteroid therapy (ACST) accelerates the maturation of the parenchymal structure, increases fluid clearance from the lungs and decreases vascular permeability [24].

Pneumothorax is more common in preterm neonates, although there are authors who found more children born at term with NP [6]. In our study, pneumothorax most commonly occurred between 34 and 36 GW.

The risk of developing pneumothorax is increased in neonates with respiratory distress syndrome (RDS), meconium aspiration syndrome (SAM), transient neonatal tachypnea (TTN), pulmonary hypoplasia, persistent pulmonary hypertension (PPHN) and neonates with applied resuscitation after birth. Lung disease is the underlying condition in 59–61% of neonates with pneumothorax [25].

In the diagnosis of pneumothorax, methods of translumination, lung ultrasound and chest X-ray are used. Lung ultrasound is used as a quick method of diagnosing pneumothorax, when treatment can be initiated, as well as for monitoring minor pneumothorax [26,27]. Although translumination and lung ultrasound are increasingly used, chest radiography is a method of diagnosis and monitoring of pneumothorax treatment. There is no doubt that ultrasound gained space both in diagnostic assistance and in medical procedures, contributing to a better prognosis and fewer complications. Compared to a simple chest X-ray, ultrasound provides better confirmation of drainage in the intercostal space after the procedure [28].

Treatment of pneumothorax depends on the severity of the clinical picture and the amount of air in the interpleural space. A smaller amount of air (partial pneumothorax) is treated conservatively, by positioning (lying on the side of the pneumothorax), while a massive pneumothorax requires urgent surgical drainage of the chest [29]. Treatment methods for a massive pneumothorax include needle aspiration (NA) and chest drainage (CD). Needle aspiration, where a needle is inserted between the ribs and removed after air aspiration has stopped, is recommended as a first-line treatment for spontaneous pneumothorax [30]. In mechanically ventilated neonates, some clinicians believe that needle aspiration will not provide adequate treatment and delay chest tube placement [31].

Complications related to emergency pleural drainage occur in 14% to 25% and can range from drain misplacement to fatal iatrogenic lesions [32]. Faced with this scenario, understanding the factors related to the incidence of complications leads to the development of measures and techniques that can reduce this rate. One of the techniques is ultrasound-guided pleural drainage in case of massive pneumothorax. Complications of thoracic drainage in neonates include chest injury as well as breast scarring, especially in female neonates [33].

Our study had some limitations. It was a single-center study, with a relatively small study sample. The risk factors for the development of NP are still unclear, and this area of research would require a prospective, well-designed study with a larger sample.

## 6. Conclusions

Significantly more children with pneumothorax were born prematurely than at term. The analysis showed that in our population the optimal threshold GW after which newborns with pneumothorax can have a good outcome was 29.5. Therefore, with adequate therapy, even premature newborns can successfully recover from pneumothorax.

## Figures and Tables

**Table 1:** Tested parameters in preterm and term neonates with pneumothorax.

Parameters		Preterm		Term	
		Frequency	Percent	Frequency	Percent
Baby gender	male	33	62.3	17	81.0
	female	20	37.7	4	19.0
Pregnancy	unifetal	43	81.1	21	100.0
	twins	10	18.9	0	0.0
Pregnancy complications	no	29	54.7	15	71.4
	GDM	4	7.5	3	14.3
	HTA	11	20.8	2	9.5
	placenta abruption	4	7.5	0	0.0
	infection	5	9.4	1	4.8
Delivery	vaginal	16	30.2	7	33.3
	CS	37	69.8	14	66.7
Baby body development	eutrophia	37	69.8	12	57.1
	hipotrophia	4	7.5	5	23.8
	hypertrophia	12	22.6	4	19.0
Pneumothorax side	right	25	47.2	10	47.6
	left	18	34.0	9	42.9
	both	10	18.9	2	9.5
Treatment	conservative	6	11.32	7	33.33
	needle aspiration	10	18.87	4	19.05
	ATD	37	69.81	10	47.62
Mod of O_2_ treatment	O_2_	9	16.98	8	38.09
	nCPAP	9	16.98	6	28.57
	MV	35	66.04	7	33.34
Cause for oxygenation	Transitory tachypnea	13	24.53	19	90.48
	asphyxia	10	18.87	2	9.52
	RSD	30	56.60	0	0.0
Surfactant	no	25	47.2	19	90.5
	yes	28	52.8	2	9.5
Corticosteroid therapy	no	27	50.9	21	100
	yes	26	49.1	0	0.0
Neonatal infection	no	32	60.4	10	47.6
	pneumonia	13	24.5	9	42.9
	sepsis	8	15.1	2	9.5
Neurological complications	no	38	71.7	17	81.0
	IVH	4	7.5	0	0.0
	convulsions	11	20.8	4	19.0
Outcome	mortality	5	9.4	1	4.8
	further therapy	5	9.4	1	4.8
	good	43	81.1	19	90.5

**Table 2:** Patient characteristics.

Parameters	Preterm	Term	Between Groups *p*
	Mean ± Standard Deviation	Mean ± Standard Deviation	
Gestational weeks (GW)	34.62 ± 4.03	38.2 ± 5.6	0.001
Baby weight	2241.3 ± 904.5	3400.5 ± 544.8	0.001
Baby length	44.9 ± 6.7	51.6 ± 2.5	0.001
Head circumference	31.6 ± 4.2	35.3 ± 1.3	0.001
Apgar score	6.5 ± 2.5	8.3 ± 0.9	0.001

## Data Availability

All data are available in the archives (database) of the Department of Neonatology, Clinic for Gynecology and Obstetrics, University Clinical Center of Serbia, Belgrade, Serbia.
